# Circular RNA circPTPRF promotes the progression of GBM via sponging miR-1208 to up-regulate YY1

**DOI:** 10.1186/s12935-022-02753-1

**Published:** 2022-11-17

**Authors:** Jiang Zhou, Chengbin Wang, Yingliang Liu, Daming Cui, Zhenlin Wang, Yang Jiang, Liang Gao

**Affiliations:** 1 Department of Neurosurgery, Shanghai Tenth People’s Hospital, Tongji University School of Medicine, 200072 Shanghai, China; 2 Department of Neurosurgery, Ningbo Medical Center Lihuili hospital, Ningbo, China; 3 Division of Experimental Neurosurgery, Department of Neurosurgery, Heidelberg University Hospital, Heidelberg, Germany

**Keywords:** Glioblastoma, Glioma stem cells, Circular RNAs, CircPTPRF, MiR-1208, YY1

## Abstract

Glioblastoma (GBM) is the most common primary malignant tumor in the brain, and its robust proliferation and invasion abilities reduce the survival time of patients. Circular RNAs (circRNAs) play an essential role in various tumors, such as regulating tumor cell proliferation, apoptosis, invasion, metastasis, and other progressive phenotypes through different mechanisms. Finding novel circRNAs may significantly contribute to the prognosis of GBM and provide the basis for the targeted therapy of GBM. In this study, we found circPTPRF is a novel circRNA that has never been studied, which was highly expressed in GBM and is closely related to poor patient prognoses. After knockdown or overexpression in glioma cell lines (U87 and LN229) and glioma stem cells (GSCs), we identified that circPTPRF could promote proliferation, invasion, and neurospheres formation abilities of GBM via in vitro and in vivo experiments. Mechanisms, miR-1208 was confirmed as a target of circPTPRF, and miR-1208 can also target the 3’UTR of YY1, and they were proved by luciferase reporter, western blotting (WB), qPCR and RNA immunoprecipitation (RIP) assays. The following rescue experiments demonstrated that circPTPRF was a miR-1208 sponge for upregulating YY1 expression to promote proliferation, invasion and neurosphere formation abilities of GBM in vitro. In conclusion, the circPTPRF/miR-1208/YY1 axis can regulate GBM progression. CircPTPRF may play an essential role in GBM diagnosis and prognostic prediction and be an important molecular target for GBM therapy.

**Supplementary information:**

The online version contains supplementary material available at 10.1186/s12935-022-02753-1.

## Introduction

Malignant tumors in the central nervous system (CNS) are extremely harmful to human health and survival. As the most frequent primary malignant tumor in CNS, glioma accounts for about 80% of all patients [1, 2]. According to the WHO Classification of CNS tumors published in 2016 [3], WHO grade IV glioma is called Glioblastoma Multiform (GBM), with the highest degree of malignancy and the shortest median survival time below 15 months [4]. Glioma stem cells (GSCs) are capable of multi-lineage differentiation and self-renewal abilities and closely associated with the progressive phenotype and progression of GBM, causing tumors’ tumorigenesis, metastasis, and chemoradiotherapy resistance [5]. Maximal surgical resection in combination with chemotherapy and radiotherapy constitutes the most common therapy [6, 7]. However, its treatment effects are still unsatisfactory [8]. Current studies have reported that various oncogenes and tumor suppressor genes play crucial roles in the development of GBM [9], and targeted therapy has been vastly performed in the clinical treatment of GBM in recent years [10, 11]. Therefore, exploring the oncogenes related to GBM has been beneficial in revealing the biological progression of GBM and contributing to a better prognosis.

Analysis of the human transcriptome shows that although more than 75% of the genome is transcribed, only 2% of the genome encodes proteins [12]. Systematic genome and transcriptome studies have revealed the profound relationship between alterations in non-coding RNAs (ncRNAs) and tumor progression [13]. Initially found in the cytoplasm of mammalian cells, circRNAs are more stable than linear RNAs because their loop structure prevents exonuclease-mediated degradation [14]. CircRNAs have been identified to interact with RNA-binding proteins and act as miRNA sponges because of the possession of abundant microRNAs (miRNAs) binding sites [15]. The miRNAs are small non-coding RNAs encoded by endogenous genes which have approximately 22 nucleotides in length [16, 17], and miRNAs bind to complementary sequences of target mRNAs by microRNA recognition elements (MRE) to down-regulate the expression of the target mRNAs and regulate the progression of various tumors [18, 19]. Consequently, circRNAs commonly perform important tumor regulatory functions as competing endogenous RNAs (ceRNA), which are the transcripts cross-regulate each other by competing for tumor-related miRNAs [20].

CircPTPRF (circBase ID: hsa_circ_0012077) is a novel circRNA located in chr1:44054401–44,054,671, and there is no relevant report on its biological function at present. The parental gene of circPTPRF is protein tyrosine phosphatase receptor type F (PTPRF). The PTP family can regulate many biological functions, including differentiation, cell cycle regulation, cellar signaling, and immune response, and its abnormal expression can promote tumor initiation [21–23].

MiR-1208 is encoded on chromosome 8q24 and is closely associated with the course of several tumors [24, 25]. MiR-1208 is rarely expressed in a range of cancer such as breast and colon cancer tissues and Burkitt’s lymphoma, suggesting that miR-1208 probably plays a biological role as a suppressor gene [26, 27]. Studies suggest that miR-1208 upregulates MAP3K2 expression to promote disease progression of hepatocellular carcinomatosis [28]. In addition, miR-1208 constitutes a tumor inhibitor directly targeting TBCK in renal cancer progression [27]. Besides, YY1 was the downstream targeted gene of miR-1208 via bioinformatics analysis. It has been reported to promote the progressive phenotypes of glioma and is related to poor prognoses in several studies [29, 30].

Through bioinformatic analysis, our study found the relationship between circPTPRF expression and the malignance of GBM. Mechanistically, we explored that circPTPRF upregulates YY1 via sponging miR-1208 to promote GBM progressive phenotypes. Our further study verified the result of bioinformatic analysis via cellular experiments based on patient-derived GSCs cell lines and intracranial tumorigenesis experiments. Therefore, this study demonstrates circPTPRF as a novel oncogene related to GBM progression. It may be a possible therapeutic target that can reduce the incidence and mortality of GBM and improve the survival time of GBM patients.

## Materials and methods

### Patient specimens and ethics

Fifty-five clinical specimens of glioma patients were collected between January 2010 to December 2014 at the Shanghai Tenth Hospital. Fifteen specimens of grade II, 20 specimens of grade III, and 20 specimens of grade IV glioma (glioblastoma) were incorporated. Ten acute brain injury patient specimens were gathered as a control group during the same period. Inclusion criteria: The patient was over 18 years old and had no other neurological diseases except glioma or GBM. The patient received surgery for the first time and did not receive preoperative radiotherapy and chemotherapy. The histological diagnoses were developed and validated by two neuropathologists based on the 2016 WHO classification guidelines. Exclusion criteria: Patients with recurrent glioma or who received radiotherapy and chemotherapy before the surgery. Clinical information of the specimens involved in the investigation is shown in Table S1. The exploration was approved by the Ethics Committee of the Shanghai Tenth Hospital, and all patients finished written informed consent. Animal experiments were performed under the supervision of the Animal Ethics Committee of Shanghai Tongji University School of Medicine.

### Cell treatment and GSCs isolation

Human glioma cells (U87, LN229) and normal human astrocyte cells (NHA) were attained from American Type Culture Collection (ATCC; Manassas, VA, USA). Isolate patient-derived GSCs, and glioma cell lines derived GSCs (U87-GSC and LN229-GSC) and perform neurosphere cultures as previously reported [31, 32]. In brief, Dulbecco’s modified Eagle’s medium (DMEM) with rh-epidermal growth factor (20 ng/mL, #E3481, Thermo Fisher Scientific), recombinant human (rh) basic fibroblast growth factor (20 ng/mL, #RP10915, Thermo Fisher Scientific) and B27 (1:50, #17,504,044, Thermo Fisher Scientific, Waltham, MA, USA) have been used to dissociate fresh clinical glioblastoma samples into single cell and culture. Then collect and culture neurospheres were in the medium mentioned above. We use self-renewal and functional assays of tumor formation in vivo to test the cancer stem cell nature of isolated GSCs. We used immunofluorescence to explore the multi-lineage differentiation capacity and the expression of stem cell markers (CD133 and nestin+) of GSCs. Clinicopathological information details are exhibited in Supplementary Table 2.

### Lentiviral vector construction and transfection

The artificial repeats and entire sequence of circPTPRF and YY1 were subcloned into pcDNA3.1 vector and pcDNA3.1 circRNA mini vector. GenePharma (Shanghai, China) engineered siRNA sequences targeted to circPTPRF and YY1 for silencing them: circPTPRF-KD1: 5′- AGGTCACAGTGAAAGTGCGCC-3′, circPTPRF-KD2: 5′- CAGGTCACAGTGAAAGTGCGC-3′. YY1-KD1: 5′- GCCTCTCCTTTGTATATTATT-3′, YY1-KD2: 5′- GCCTCTCCTTTGTATATTATT-3′. Obtain the miR-1208 mimic, inhibitor, and their negative controls from Thermo Fisher Scientific (Assay ID: MC13539, AM13539 and AM17010). The miRNA inhibitor is a small, chemically modified single-stranded RNA molecule that specifically binds to and inhibits endogenous miRNA molecules and enables miRNA functional analyses through the downregulation of miRNA activities. Each cell was examined for resistance to puromycin (#OGS269, Sigma, Santa Clara, CA, USA) for 15 days at a 10 µg/ml content after transfection. Mimics and inhibitor of miR-1208 came from GenePharma as well.

### Quantitative real-time PCR (qRT‐PCR)

Total RNA was extracted utilizing TRIzol reagent (#15,596,018, Invitrogen, Carlsbad, CA, USA). M-MLV reverse transcriptase (#28,025,021, Invitrogen) or miScript reverse transcription kit (#205,311, QIAGEN, Hilden, Germany) were used for synthesizing complementary DNA (cDNA). Calculate the relative expression of circPTPRF, PTPRF mRNA, miR-1208 and YY1 based 2^−∆∆Ct^ approach. Use glyceraldehyde-3-phosphate dehydrogenase (β-actin) to be the inside reference. PCR primer pair sequences were below: circPTPRF, forward 5′ - GTCCTGGAGCTCAGCAATGT-3′ and reverse 5′ - AGGGATGGAGAAACGAGGAG-3′; YY1, forward 5′ - ACGGCTTCGAGGATCAGATTC-3′ and reverse 5′ - TGACCAGCGTTTGTTCAATGT-3′; YY2, forward 5′ - ATGGCCTCCAACGAAGATTTC-3′ and reverse 5′ - TCCGTCGGAATGTCCTCCATA-3′; β-actin, forward 5′ - CATGTACGTTGCTATCCAGGC-3′ and reverse 5′ - CTCCTTAATGTCACGCACGAT-3′.

### RNase R treatment

Incubate 10 µg RNA extracted from the patient-derived cell lines by RNase R (4U/µg; #RNR07250, Epicentre Biotechnologies, Madison, WI, USA) or not for one hour at 37 °C. Then, use qRT-PCR to detect the relative expression of circPTPRF and PTPRF mRNA.

### WB

Perform WB as depicted previously [33]. A cell protein extraction kit (#KGP3100, KeyGen Biotechnology, Nanjing, China). BCA protein quantification kit (#KGP902, KeyGen Biotechnology) was employed to determine its contents. Transfer 40 µg protein specimens to polyvinylidene fluoride films (#88,518, Thermo Fisher Scientific) after sodium dodecyl sulfate-polyacrylamide gel electrophoresis and then block them with fat-free milk. The primary antibodies against YY1 (1:2000, #ab109237, Abcam, Cambridge, UK) and β-actin (1:2000, #66009-1-Ig, ProteinTech, Chicago, IL, USA) were used. Use IMAGE J software (National Institutes of Health, Bethesda, MD, USA) and ECL kits (#P0018S, Beyotime Biotechnology, Beijing, China) for quantification.

### Immunofluorescence

Perform immunofluorescence as depicted previously [34]. Fix, permeabilize and block Cells employing 4% paraformaldehyde (#P6148, Sigma, Santa Clara, CA, USA), 0.5% Triton X-100 (#SH-0938, Kaissy Biotechnology, Beijing, China), and 5% BSA (#15,561,020, Invitrogen), incubate them with primary antibody overnight at 4 °C before FITC- or rhodamine-conjugated secondary antibody. Use the antibodies against β III tubulin (1:1000, #ab7751, Abcam), GFAP (1:1000, #ab4674, Abcam), nestin+ (1:500, #ab18102, Abcam) and CD133 (1:1000, #ab222782, Abcam). DAPI (#D9542, Sigma) was used for staining the nuclei. Use a laser scanning confocal microscope (IX71, Olympus, Tokyo, Japan) for detecting and photographing.

### MTS proliferation assay

Use the CellTiter 96 Aqueous non-radioactive cell proliferation detection kit (#G5421, Promega, Madison, WI, USA) for evaluating GCS proliferation. Briefly, digest and culture GSCs in 96-well plates at 1 × 10^3^ cells/well for 24, 48, 72, 96, or 120 h. The absorption was detected by an ultraviolet spectrophotometer (Thermo Fisher Scientific) at 495 nm.

### 5-Ethynyl-2’-deoxyuridine (EdU) proliferation assay

Perform EdU assays as previously depicted [35]. In brief, seed cell lines in 24-well plates at 1 × 10^5^ cells/well for 24 h. Then, add EdU reagent (#C0078L, Beyotime Biotechnology) at 37 °C for two hours. Fix the cells adopting paraformaldehyde. Finally, the EdU-positive cells were visualized using a laser scanning confocal microscope (Olympus).

### Transwell assay

Seed cells in the upper chamber (8 μm) coated with 100 µl Matrigel (#356,234, BD Biosciences, CA, USA) at a density of 5 × 10^4^ cells/well. Fill the lower chamber employing DMEM supplemented with 20% FBS. Incubation was set at 37◦C with 5% CO2 for 24 h. Then, fix cells in the lower chamber with 4% paraformaldehyde and stain them with crystal violet. Count the stained cell quantity under an inverted microscope.

### Limiting dilution neurosphere formation assay

Assess the self-renewal ability of GSCs through the neurosphere formation assay as previously reported [31]. Briefly, dissociate and seed GSCs into 24-well plates and incubate them in a fresh medium for 7 days. Count the relative size and quantity with an optical microscope (Olympus). In vitro limiting dilution approach, seed GSCs at a gradient of 1, 10, 20, 30, 40 or 50 cells/well into 96-well plates for seven days. Later, calculate neurosphere quantity in all wells applying the Absolute Limiting Dilution Analyses (ELDA) (http://bioinf.wehi.edu.au/software/elda) [36].

### Luciferase activity analysis

Perform luciferase reporter assays (LRAs) as previously depicted [37]. In brief, clone mutant-type YY1, wild-type YY1, mutant-type circPTPRF and wild-type circPTPRF into the empty pmiRGLO luciferase reporter vector (Promega). Then, use Dual-Luciferase Reporter Assay System (#E1910, Promega) to test luciferase activity.

### RNA immunoprecipitation (RIP) assay

Perform RIP assay employing the EZ-Magna RIP RNA-binding Protein Immunoprecipitation Kit (#17–701, Millipore, Darmstadt, Germany). Lyse GSCs in various conditions applying RIP lysis buffer and magnetic beads conjunct with antibodies against Ago2 and antibodies against IgG as a negative control. Isolate the immunoprecipitated RNAs after incubating, adopting proteinase K (#ST532, Beyotime Biotechnology). Lastly, use qRT-PCR to detect the expressions of circPTPRF, miR-1208 and YY1.

### Xenograft experiments

Inject transfected GSCs (5 × 10^4^ cells per mice) orthotopically into the brains of 6-week-old female BALB/c nude mice (Beijing Vital River Laboratory Animal Technology, Beijing, China). After being determined with a stereotaxic apparatus, the injection point was 2 mm lateral and 2 mm anterior to the bregma. Mice have been observed daily for neurological symptoms or death, and the tumor volume was determined as V = (D × d^2^)/2, D represents the longest diameter, and d represents the shortest diameter of the tumor. When neurological symptoms were observed, sacrifice mice employing cervical spine dislocation and collect the brains for analyses according to the previously identified method [37], perform each animal experiment following the Animal Care Committee guidelines of Shanghai Tong Ji University School of Medicine.

### Immunohistochemistry (IHC)

Perform IHC of mice xenograft tumor specimens as previously depicted [38]. Briefly, paraformaldehyde was used to fix fresh mice tumor tissue. The samples were dehydrated by ethanol, permeabilized with xylene, embedded in paraffin wax, and sectioned at 4 μm. Then xylene was used to deparaffinized, followed by rehydration and deionized water hydration to remove endogenous peroxidase. Slides got microwave treatment in the sodium citrate buffer for 15 min to retrieve antigen. 10% normal goat serum was added to the block for another 30 min at room temperature before incubating primary antibodies against YY1, ki-67 and secondary antibodies (Abcam). The immunostaining was tested by adopting 3,3′-diaminobenzidine (Sigma) and counterstained by applying hematoxylin. Use an optical microscope to image the sections and take photos. (Olympus)

### Bioinformatic analysis

Obtain the data of circRNA expression in gliomas from Gene Expression Omnibus (GEO) data sets GSE146463. Use Starbase (starbase.sysu.edu.cn), Targetscan (http://www.targetscan.org/vert_71/), miRWalk (http://mirwalk.umm.uni-heidelberg.de) and MiRDB (http://mirdb.org) for predicting the binding sites between miR-1208 and circPTPRF or YY1.

### Statistic analyses

Use SPSS 22.0 software (IBM, Armonk, NY, USA) to perform statistical analyses. Repeat each experiment over three times, and express the outcomes as average ± standard error. Test comparison between groups through the t-test and chi-square test. Use the Log-rank test and Kaplan-Meier analyses for analyzing survival rates. Use Pearson’s correlation analyses to analyze the correlation among circPTPRF, miR-1208 and YY1. Define statistic significance as a P-value < 0.05. GraphPad Prism 8.0 (GraphPad Software Inc, San Diego, C.A, USA) was used to plot figures.

## Results

### CircPTPRF is upregulated in glioma tissues and correlated with poor prognoses

**Fig. 1:**
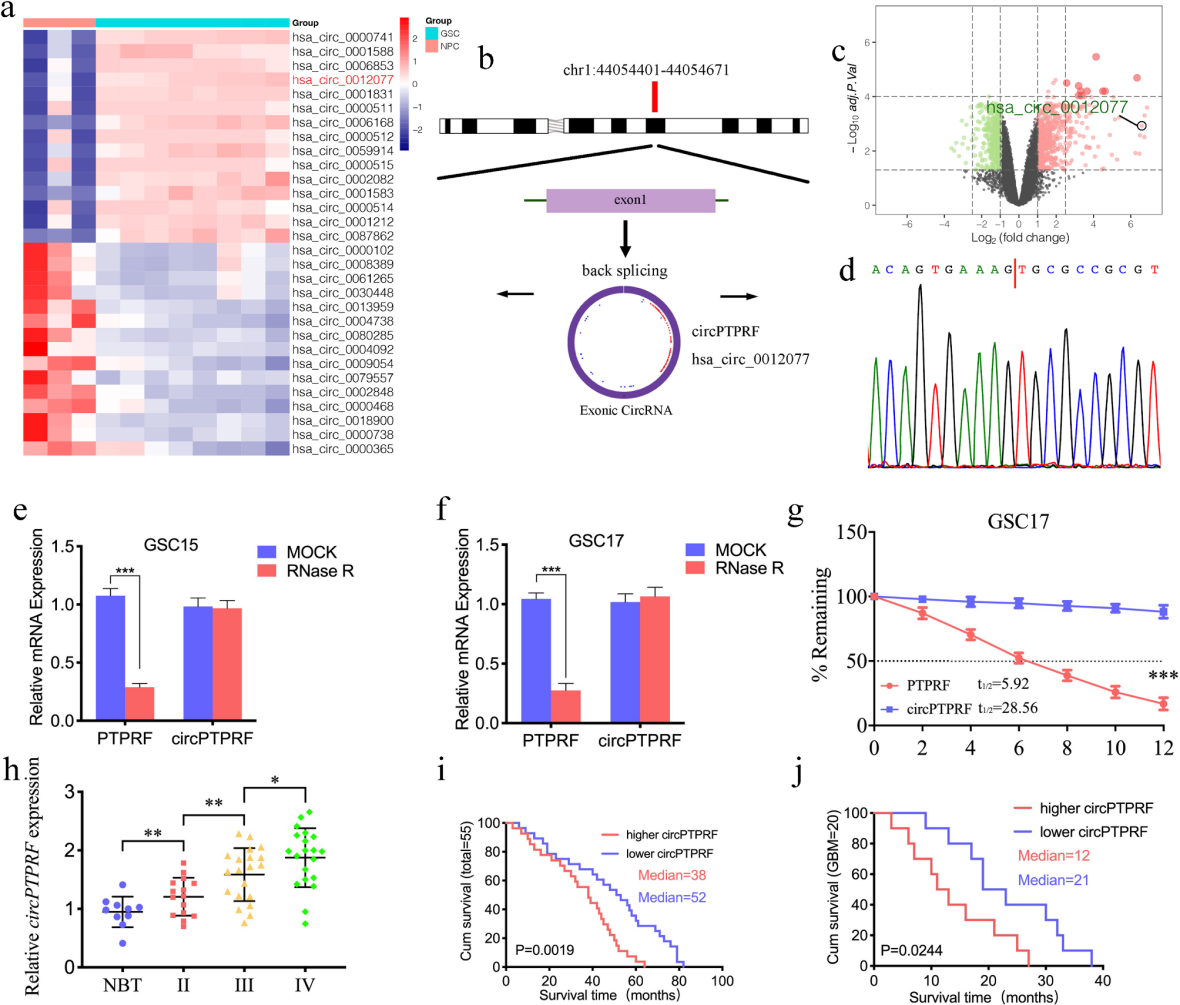
CircPTPRF is up-regulated in glioma tissues and correlated with the poor prognosis. a: Heatmap showed circPTPRF expression was the fourth overexpressed circRNAs in glioma stem cells (GSCs, n = 8) than in neural progenitor cells (NPCs, n = 3) based on GEO datasets GSE109569. b: Schematic diagram illustrating the circular structure of circPTPRF. c: The Volcano map showed the expression of circPTPRF in GSCs (n = 8) compared with NPCs (n = 3). d: Sanger sequencing validated the specific junction of circPTPRF. e, f: qPCR analysis of PTPRF mRNA and circPTPRF in GSC15 and GSC17 treated with Rnase R g: half-life detection confirmed the circular characteristics of circPTPRF. h: circPTPRF expression was correlated with glioma WHO grades. i, j: Kaplan-Meier analysis of all patients (i) or GBM patients (j) based on higher circPTPRF expression. All data are shown as the mean ± SD (three independent experiments). *P < 0.05; **P < 0.01; ***P < 0.001

We firstly found that the expression of circPTPRF was strongly higher in GSCs than in neural progenitor cells (NPCs) according to GEO dataset GSE146463 (Fig. 1a, c). Hsa_circ_0012077 (circPTPRF) was the fourth overexpressed circRNAs in GSCs via limma analysis. CircPTPRF comes from transcript 1 of the PTPRF gene (chr1: 44,054,401–44,054,671), and its loop structure is shown in Fig. 1b. According to UCSC, SLC6A9, ST3GAL3, TIE1 and CDC20 are the neighboring genes for circPTPRF. Use sanger sequencing to verify the particular junction of circPTPRF (Fig. 1d). Perform RNase R assays frequently to confirm RNAs’ circular structure since linear RNAs can be degraded with short 3′-tails. In contrast, circRNAs can not be degraded [39]. PTPRF mRNA expression was obviously lower in GSC15 and GSC17 cell lines after RNase R treatment, while circPTPRF was unaffected, revealing that circPTPRF is more resistant to RNase R digestion (Fig. 1e, f). Furthermore, the RNA stability assay illustrated that the half-life time of circPTPRF exceeded PTPRF (Fig. 1g).

Then detect the expression of circPTPRF in 55 glioma patients via qPCR assays. The results showed circPTPRF expression was upregulated with a higher WHO grade (Fig. 1h). After that, Kaplan-Meier survival analyses revealed that the average survival time of patients featuring lower circPTPRF expression obviously exceeded those featuring higher expression in both the total and GBM groups (qPCR quantification, Cutoff: median, Fig. 1i, j).

### Over-expression of circPTPRF enhanced proliferation and invasion of GBM in vitro

Patient-derived GSCs were further extracted from fresh clinical GBM samples for further studies, and then six GSCs were chosen in the best growth station. Immunofluorescence staining demonstrated the expression of the stem cell markers CD133 + and Nestin + in the separated neurospheres (Sup. Figure 1a). Meantime, the multi-lineage differentiation capability of GSCs was also exhibited in Supplementary Fig. 1b. Then, qPCR was adopted for determining circPTPRF expression in six cell lines, with GSC15 featuring the lowest expression and GSC17 the highest (Sup. Figure 1c).

**Fig. 2:**
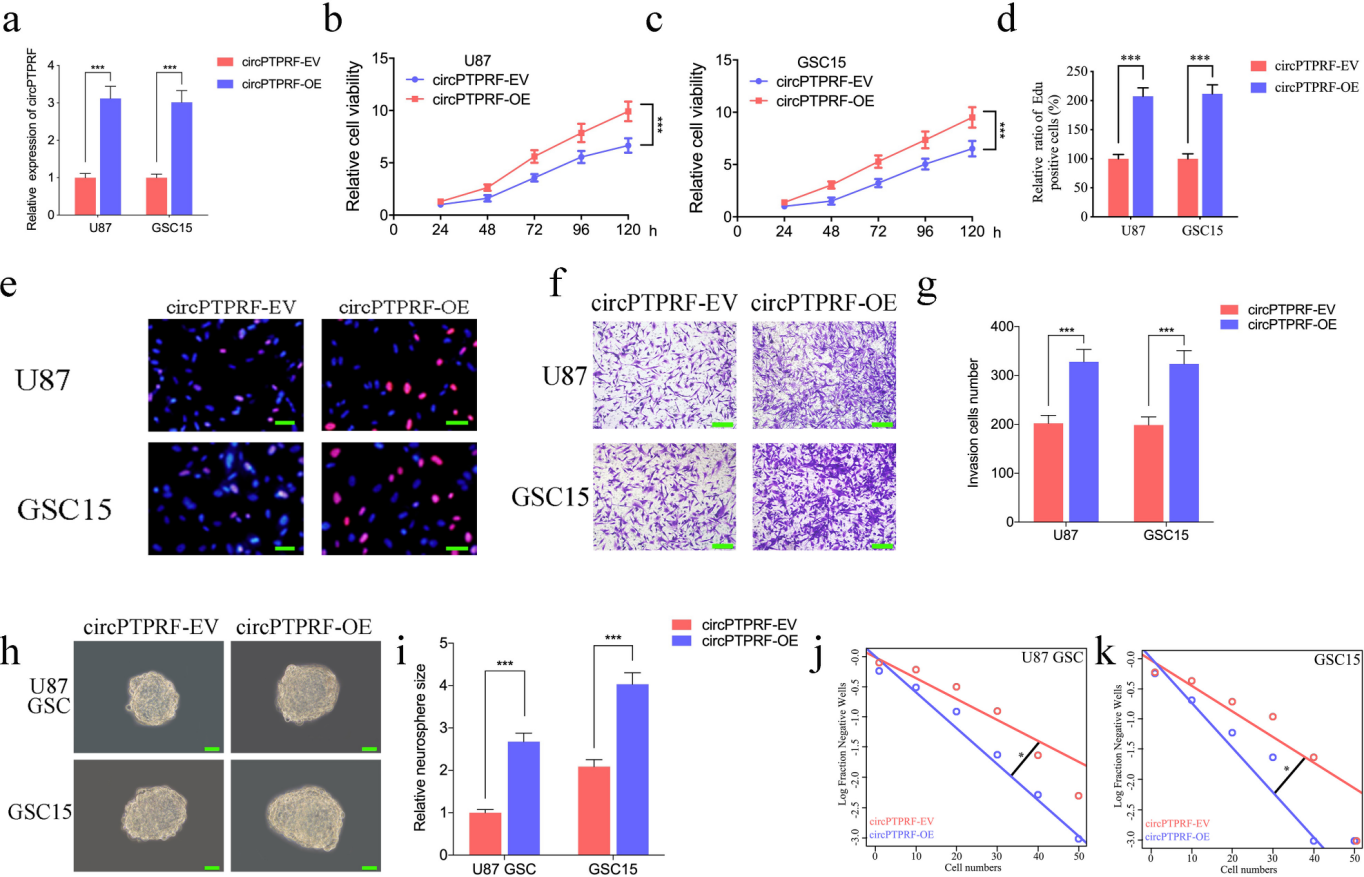
Overexpression of circPTPRF promoted proliferation and invasion of glioma in vitro. a: The expression of circPTPRF in U87 and GSC15 after transfection of the circPTPRF overexpression plasmids measured by qPCR. b, c: CircPTPRF overexpression increased the proliferation of U87 and GSC15 in MTS assays. d, e: Proliferation of tumor cells was promoted after circPTPRF overexpression as measured by Edu assays. Scale bar = 50 μm. f, g: Representative images of transwell assay to demonstrate invasion capacity can be promoted after circPTPRF overexpression. Scale bar = 50 μm. h-k: Representative images of neurospheres (k, l) and extreme limiting dilution assays (m, n) showed tumor formation rate up-regulated after circPTPRF overexpression in U87-GSCs and GSC15. Scale bar = 50 μm. All data are shown as the mean ± SD (three independent experiments). *P < 0.05; **P < 0.01; ***P < 0.001

To find the function of circPTPRF in GBM, we conducted gain-of-function assays via designing lentiviral-based circPTPRF overexpressed plasmid, which was used to infect GSC15 and U87. qPCR identified circPTPRF overexpression was the best (Fig. 2a). First, we confirmed that the absorbance values of cell lines obviously exceeded those of controls after circPTPRF overexpression by MTS assays (Fig. 2b, c), verifying circPTPRF over-expression up-regulated the viability of cell lines. Then, Edu assays were used to explore the role of circPTPRF in tumor proliferative capability. The proportion of Edu-positive cells in over-expressed cell lines exceeded those in the empty vector group (EV), which indicated tumor proliferative capability was enhanced after circPTPRF over-expression (Fig. 2d, e). Using transwell assays to measure tumor cell line invasion, we identified larger metastatic potential in circPTPRF overexpression compared to EV groups (Fig. 2f, g). Furthermore, the relative size of neurospheres that took shape per unit time after circPTPRF over-expression was more significant than those in the EV groups via neurosphere formation assays (NFAs) (Fig. 2h, i). ELDA also illustrated that circPTPRF over-expression raised the tumor formation rate (Fig. 2j, k). All the results above showed that higher circPTPRF expression could promote the progressive phenotypes of GBM.

### CircPTPRF knockdown suppressed the proliferation and invasion of GBM in vitro

**Fig. 3:**
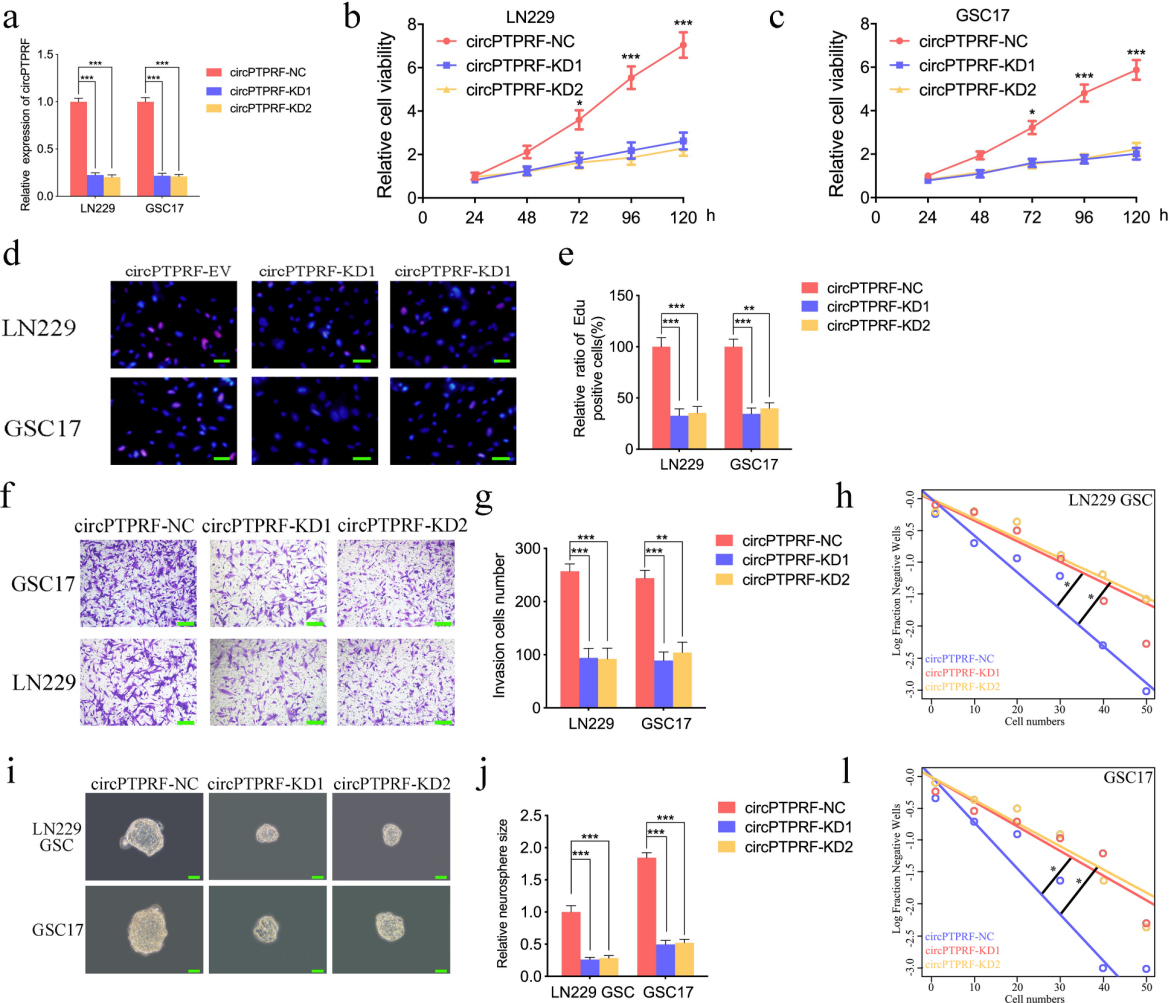
CircPTPRF knockdown suppressed the proliferation and invasion of glioma in vitro. a: The expression of circPTPRF in LN229 and GSC17 after transfection of circPTPRF -KD1, circPTPRF -KD2 or negative control measured by qPCR. b, c: CircPTPRF knockdown significantly decreased the proliferation of LN229 and GSC17 in MTS assays. d, e: CircPTPRF knockdown can greatly reduce the proliferative capacity of LN229 and GSC17 measured by Edu assays. Scale bar = 50 μm. f, g: Representative images of transwell assay to demonstrate invasion capacity can be suppressed after circPTPRF knockdown. Scale bar = 50 μm. h-k: Neurospheres formation assays and extreme limiting dilution assays showed that the tumor formation rates decreased after circPTPRF knockdown in LN229-GSCs and GSC17. Scale bar = 50 μm. All data are shown as the mean ± SD (three independent experiments). *P < 0.05; **P < 0.01; ***P < 0.001

We furtherly investigate whether circPTPRF knockdown can inhibit the progressive phenotypes of GBM via siRNA. Use qPCR to confirm that circPTPRF knockdown efficiency was satisfactory in LN229 and GSC17 (Fig. 3a). MTS assays found an apparent drop in absorbance values after circPTPRF knockdown, showing the viability of tumor cells was obviously down-regulated (Fig. 3b, c). Edu assays identified circPTPRF knockdown could reduce Edu-positive cell proportion (Fig. 3d, e). Transwell assays illustrated that invasion of LN229 and GSC17 were inhibited after circPTPRF knockdown (Fig. 3f, g). NFAs exhibited that the smaller neurosphere relative size was decreased in circPTPRF knockdown groups (Fig. 3h, i). Besides, the ELDA assays demonstrated that circPTPRF knockdown strongly decreased neurospheres’ formation rate (Fig. 3j, k). The results above verified that circPTPRF knockdown could suppress the progressive phenotypes of GBM.

### CircPTPRF overexpression reversed proliferation and invasion suppression of GBM in vitro caused by circPTPRF knockdown

We furtherly performed rescue assays to confirm the promoting proliferation and invasion effects of GSCs via overexpressed circPTPRF expression in circPTPRF knockdown LN229 and GSC17. MTS assays showed the absorbance values in circPTPRF knockdown groups were obviously reversed and up-regulated after circPTPRF overexpression, showing the viability of tumor cells was obviously up-regulated (Sup. Figure 2a, b). Edu assays identified circPTPRF knockdown could reduce Edu-positive cell proportion, while the Edu-positive cell proportion was up-regulated after circPTPRF overexpression (Sup. Figure 2c, d). Transwell assays showed that circPTPRF knockdown decreased invasion cell numbers of LN229 and GSC17 while up-regulated after circPTPRF overexpression (Sup. Figure 2e, f). In conclusion, circPTPRF overexpression could reverse the proliferation and invasion suppression of GBM in vitro caused by circPTPRF knockdown. CircPTPRF can promote the proliferation and invasion of GBM in vitro.

### MiR-1208 could bind with circPTPRF and mediate the function of GBM cells

**Fig. 4:**
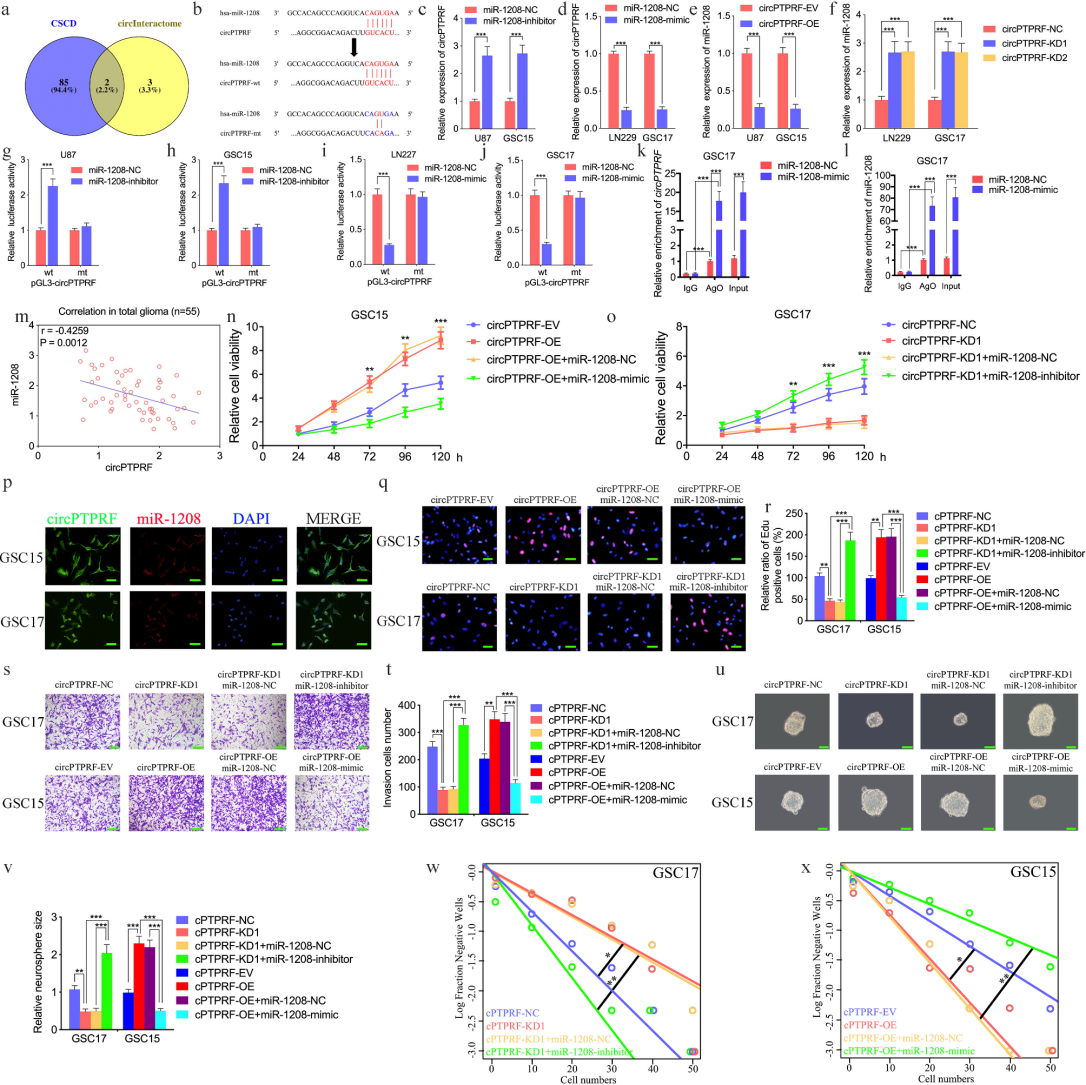
MiR-1208 could bind with circPTPRF and mediate the function of GBM cells. a: Datasets analysis explored potential miRNAs related to circPTPRF. b: The predicted binding site between circPTPRF and miR-1208. c, d: miR-1208 inhibitor treatment up-regulated the expression of circPTPRF while mimic treatment down-regulated circPTPRF expression measured by qPCR. e, f: The expression of miR-1208 was down-regulated after circPTPRF overexpression while circPTPRF knockdown up-regulated miR-1208 expression was measured by qPCR. g-j: The luciferase reporter assays showed that miR-1208 inhibitor (g, h) or mimic (i, j) altered the luciferase promoter activities of circPTPRF.k, l: CircPTPRF and miR-1208 were effectively pulled down by anti-AGO2 antibodies compared to IgG, and both were enriched after miR-1208 mimic treatment in GSC17. m: The correlation between the miR-1208 and circPTPRF expression levels as measured by qPCR in 55 glioma clinical samples. n, o: MTS assays showed that CircPTPRF transfection of overexpression plasmids or si-circPTPRF affected GSCs viability and was reversed by a miR-1208 mimic or inhibitor treatment, respectively. p: FISH assays showed the subcellular localization of circPTPRF and miR-1208. q, r: The EDU assay showed that CircPTPRF transfection of overexpression plasmids or si- circPTPRF affected GSCs proliferation capacity and was reversed by a miR-1208 mimic or inhibitor treatment, respectively. Scale bar = 50 μm. s, t: The transwell assay showed that CircPTPRF transfection of overexpression plasmids or si- circPTPRF affected GSCs invasion capacity and was reversed by a miR-1208 mimic or inhibitor treatment, respectively. Scale bar = 50 μm. u-x: In the neurosphere formation assays and extreme limiting dilution assays, circ circPTPRF transfection of overexpression plasmids or si-circASPM affected neurosphere growth capacity in GSCs and was reversed by a miR-130b-3p mimic or inhibitor treatment, respectively. Scale bar = 50 μm. All data are shown as the mean ± SD (three independent experiments). *P < 0.05; **P < 0.01; ***P < 0.001

As we mentioned before, circRNAs frequently exert their tumor promotion functions via miRNAs sponging to regulate target genes [2], so we searched the CSCD and circInteractome dataset to explore the targeted miRNAs of circPTPRF (Fig. 4a). Two intersections were found as miR-1208 and miR-647, of which miR-647 has been reported to play a pro-glioma effect [40], while there was no study about miR-1208 in glioma and GBM and miR-1208 was chosen for further exploration. The miR-1208 binding site on circPTPRF was illustrated in Fig. 4b. We subsequently used qPCR assays and found that the expression of circPTPRF was increased in U87 and GSC15 after administering the miR-1208 inhibitor, while the opposite results were obtained in LN229 and GSC17 after the treatment of the miR-1208 mimic (Fig. 4c, d). The relative expression of miR-1208 was also negatively regulated through circPTPRF contents after knockdown and overexpression of circPTPRF. (Fig. 4e, f). We further conducted LRAs and identified the raised luciferase capability of wild-type circPTPRF in U87 and GSC15 after administering the miR-1208 inhibitor (Fig. 4g, h). Conversely, the administration of miR-1208 mimics down-regulated the luciferase capability of wild-type circPTPRF in LN229 and GSC17 (Fig. 4i, j). As the RNA-induced silencing complex (RISC) is an essential pathway for miRNAs to perform their biological functions, and the Ago 2 (AGO2) protein constitutes an integral part of RISC [41], anti-AGO2 RIP assays were performed to verify if miR-1208 and circPTPRF are co-enriched in RISC. The outcomes exhibited that anti-AGO2 antibodies were pulled down in circPTPRF and miR-1208 compared with IgG. Moreover, circPTPRF and miR-1208 exhibited obvious enrichment after administering miR-1208 mimic compared with NC groups (Fig. 4k, l). Then, the expression of miR-1208 and circPTPRF in our 55 clinical samples of glioma patients was detected via qPCR, and the result showed negative correlations between these two molecules (Fig. 4m). Furtherly, we detected the subcellular localization of circPTPRF and miR-1208 were detected by the fluorescence in situ hybridization assays. The results showed that both of them were located in the cytoplasm (Fig. 4p). In conclusion, miR-1208 can bind to circPTPRF and negatively regulate its expression.

Then similar experiments in vitro were performed to determine whether miR-1208 could mediate circPTPRF induced phenotypes of GBM. MTS assays showed that circPTPRF overexpression in GSC15 could increase tumor proliferation. However, the miR-1208 mimic treatment led to significantly weaker tumor proliferation (Fig. 4n). Respectively, the tumor suppression effect induced through circPTPRF knockdown was utterly reversed after miR-1208 inhibitor remedy in GSC17 (Fig. 4o). Edu assays showed Edu-positive cell proportion was up-regulated after circPTPRF overexpression and decreased substantially after miR-1208 mimic remedy. In contrast, Edu-positive cell proportion decreased after circPTPRF knockdown and reversed on a large scale after miR-1208 inhibitor treatment (Fig. 4q, r). Similar effects were also found in transwell assays. MiR-1208 mimic or inhibitor treatment could also reverse the enhancing or suppressing invasion ability of circPTPRF over-expression or knockdown in glioma cell lines, respectively (Fig. 4s, t). NFAs and ELDA also exhibited a similar trend (Fig. 4u-x). The results strongly imply that miR-1208 might mediate the promoting function of circPTPRF on GBM.

### MiR-1208 could bind with 3’-UTR of YY1

**Fig. 5:**
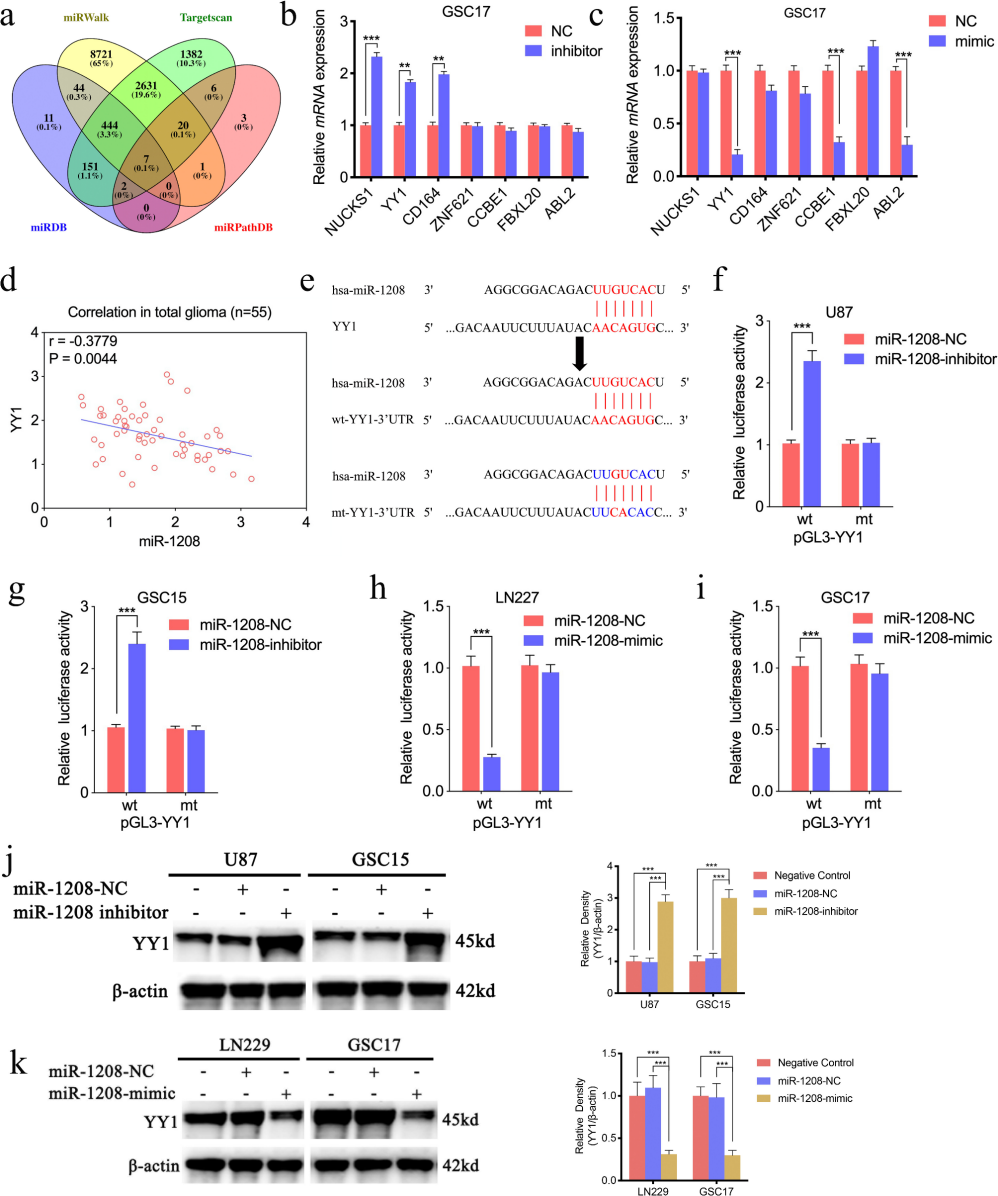
MiR-1208 could bind with 3’-UTR of YY1. a: Datasets analysis explored potential target gene-related to miR-1208. b, c: qPCR assays were performed to detect the expression of these 7 mRNAs in GSC17 after miR-1208 inhibitor or mimic treatment. d: The correlation between the miR-1208 and YY1 expression levels as measured by qPCR in 55 glioma clinical samples. e: Schematic diagram of the putative miR-1208 binding site in the 3′-UTR of YY1. f-i: The luciferase reporter assays showed that miR-1208 inhibitor (f, g) or mimic (h, i) altered the luciferase promoter activities of YY1. j, k: The decreased or increased expression of YY1 in cell lines after miR-1208 inhibitor or mimic treatment, determined by western blotting and gray quantitative analysis. All data are shown as the mean ± SD (three independent experiments). *P < 0.05; **P < 0.01; ***P < 0.001

We furtherly explored the downstream targeted oncogenes after illustrating miR-1208 sponging of circPTPRF. There are 7 targeted genes of miR-1208 according to 4 datasets as miRWalk, Targetscan, miRDB and miRPathDB (Fig. 5a). They are NUCKS1, YY1, CD164, ZNF621, CCBE1, FBXL20 and ABL2. Therefore, qPCR assays were conducted to detect the expression of these 7 genes after miR-1208 inhibitor or mimic treatment in GSC17. The results demonstrated that YY1 was the best candidate gene that can be regulated by miR-1208 (Fig. 5b, c). The expression of miR-1208 and YY1 was also detected in our 55 glioma clinical samples via qPCR. The result showed negative correlations between these two molecules (Fig. 5d). Figure 5e illustrates that miR-1208 can bind to the 3′-UTR of YY1. Therefore, we designed LRAs and identified that the administration of the miR-1208 inhibitor obviously raised the luciferase activity of the YY1-wt group (Fig. 5f, g). While the luciferase activity of YY1-wt dropped after miR-1208 mimic administration (Fig. 5h, i). WB exhibited a remarkable up-regulation in YY1 expression after administration of miR-1208 inhibitor in U87 and GSC15, and an opposite trend was obtained after administration of miR-1208 mimic in LN229 and GSC17 (Fig. 5j, k). Moreover, YY2 is a single exon retrotransposed gene that may bind the identical consensus sequences and even antagonize YY1, so we detected YY2 expression in GSC15 and GSC17 after miR-1208 mimic or inhibitor treatment, respectively. Both qPCR and WB showed there were almost no changes in YY2 after miR-1208 mimic or inhibitor treatment (Sup. Figure 3).

### CircPTPRF promoted proliferation and invasion of GBM via miR-1208 sponging to regulate YY1 expression

**Fig. 6:**
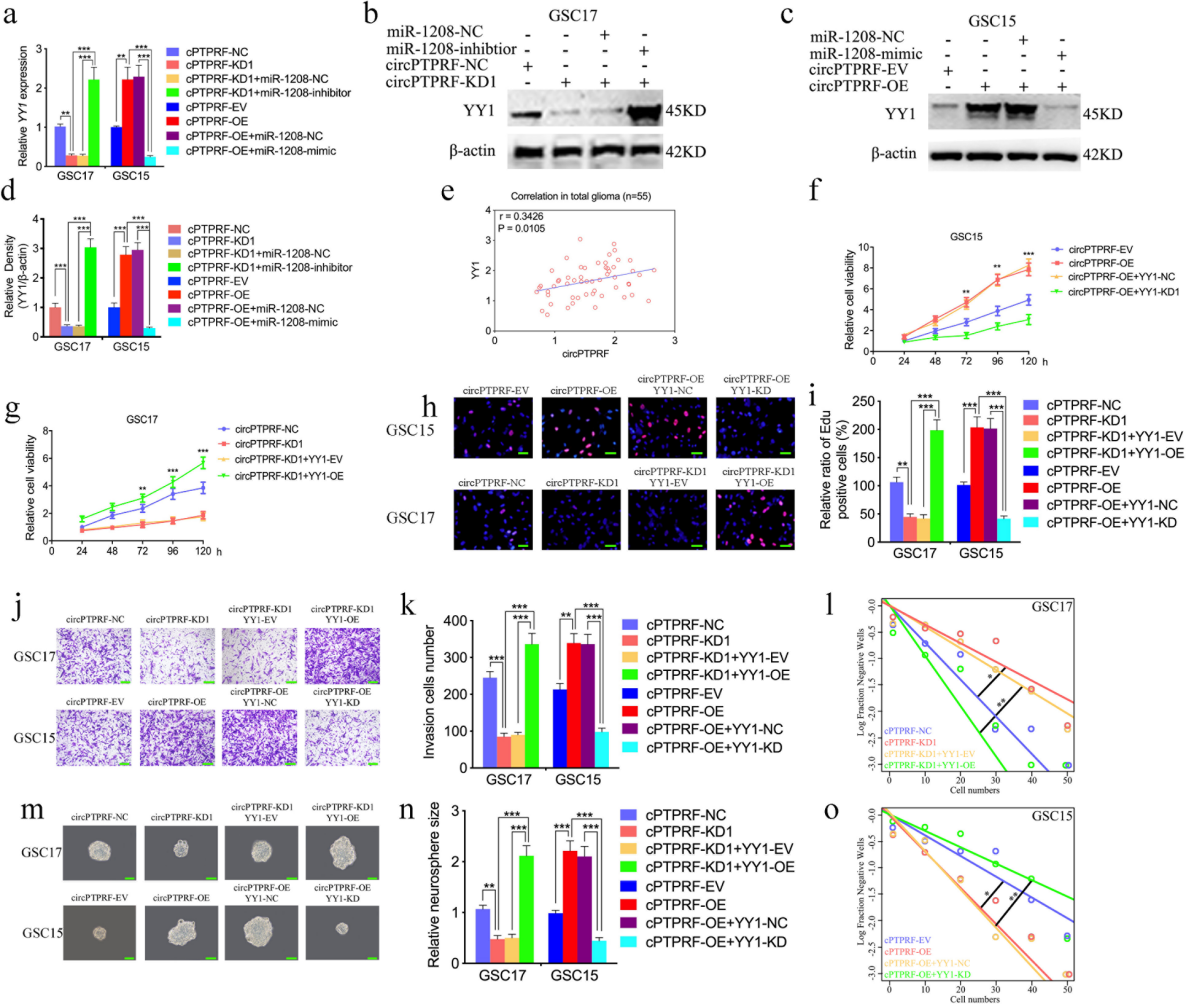
CircPTPRF mediated glioma cells by acting as ceRNA of miR-1208 to regulate YY1 expression. a-d: The down-regulated or up-regulated expression of YY1 in GSCs induced by circPTPRF knockdown or overexpression was reversed by miR-1208 inhibitor or mimic treatment, determined by qPCR, western blotting and gray quantitative analysis. e: The correlation between the circPTPRF and YY1 expression levels as measured by qPCR in 55 glioma clinical samples. f, g: MTS assays showed that the GSCs viability regulated by circPTPRF knockdown or overexpression treatment was reversed by YY1 overexpression or knockdown, respectively. h, i: The EDU assays showed that the GSCs proliferation regulated by circPTPRF knockdown or overexpression treatment were reversed by YY1 overexpression or knockdown, respectively. Scale bar = 50 μm. j, k: The transwell assays showed that the GSCs invasion capacity regulated by circPTPRF knockdown or overexpression treatment was reversed by YY1 overexpression or knockdown, respectively. Scale bar = 50 μm. l-o: The neurosphere formation assays and extreme limiting dilution assays showed that the circPTPRF transfection of overexpression plasmids or si-circPTPRF affected neurosphere growth capacity in GSCs and was reversed by YY1 overexpression or knockdown, respectively. Scale bar = 50 μm. All data are shown as the mean ± SD (three independent experiments). *P < 0.05; **P < 0.01; ***P < 0.001

We furtherly designed and conducted various rescue experiments to find if circPTPRF upregulates YY1 expression in GBM via miR-1208 sponging to YY1. qPCR and WB showed circPTPRF overexpression could upregulate YY1 expression and miR-1208 mimic treatment can reverse the upregulating effects, while the opposite results were found in circPTPRF knockdown combined with miR-1208 inhibitor treatment (Fig. 6a-d). The expression of circPTPRF and YY1 was detected in our 55 glioma clinical samples through qPCR. The result showed positive correlations between these two molecules (Fig. 6e). MTS assays (Fig. 6f, g), EDU assays (Fig. 6h, i), transwell assays (Fig. 6j, k), neurospheres formation assays (Fig. 6m, n) and ELDA (Fig. 6L, o) illustrated that circPTPRF knockdown in GSC17 obviously inhibited the viability, proliferation capability, invasion capacity and formation rate of neurospheres in GSC17. However, after YY1 overexpression, the above inhibition effects were all reversed. The opposite results were also found in circPTPRF overexpressed combined with YY1 knockdown treatment in GSC15. In summary, circPTPRF is key to GBM progression via miR-1208 sponging to regulate YY1 expression.

### CircPTPRF promoted GSCs tumorigenesis in vivo

**Fig. 7:**
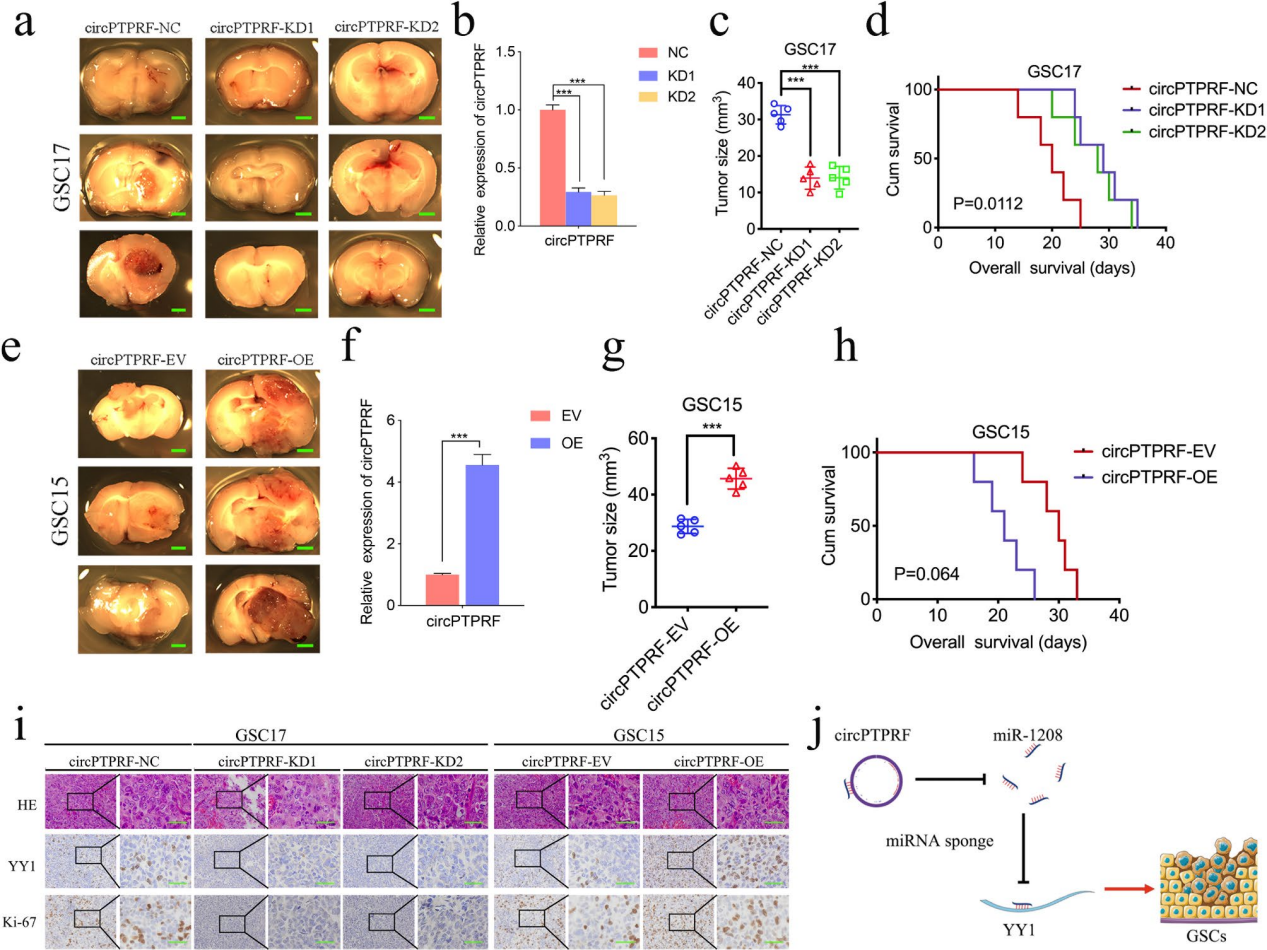
CircPTPRF promoted GSCs tumorigenesis in vivo. a, c: CircPTPRF knockdown inhibits tumor growth in vivo. Scale bar = 1 mm. b: The expression of circPTPRF in circPTPRF knockdown tissues. d: Kaplan-Meier survival curve of circPTPRF knockdown in GSC17. e, g: CircPTPRF overexpression enhances tumor growth in vivo. Scale bar = 1 mm. f: The expression of circPTPRF in circPTPRF overexpression tissues. h: Kaplan-Meier survival curve of circPTPRF knockdown in GSC15. i: Representative immunohistochemical staining showed the changes in YY1 and ki-67 staining after circPTPRF overexpression and knockdown. Scale bar = 50 μm. j: Schematic diagram illustrated that circPTPRF promotes the progression of GBM via sponging miR-1208 to up-regulate YY1. All data are shown as the mean ± SD (three independent experiments). *P < 0.05; **P < 0.01; ***P < 0.001

We further performed a tumor xenograft model to gain additional reference to the function of circPTPRF in vivo. We first detected circPTPRF expression in those mice bearing tumor tissues. CircPTPRF showed lower expression in circPTPRF knockdown groups while higher expression in circPTPRF overexpression groups (Fig. 7b, f). Then we found that circPTPRF knockdown led to significantly smaller tumor size (Fig. 7a, c) and a remarkably longer median survival time in GSC17 (Fig. 7d). In contrast, circPTPRF overexpression obviously advanced intracranial tumor development in GSC15 (Fig. 7e, g) and decreased median survival time in GSC15 (Fig. 7h). Via immunohistochemical staining and H&E staining, we identified the expression and staining intensities of YY1 and ki-67 exceeded in the control group after circPTPRF overexpression. Opposite outcomes were gained in the knockdown group (Fig. 7i). The schematic diagram illustrated that circPTPRF promotes the progression of GBM via sponging miR-1208 to up-regulate YY1 (Fig. 7j). The above results verified that circPTPRF promotes GBM tumorigenesis also in vivo.

## Discussion

GBM, or grade IV glioma, is the most malignant pathological type, and the prognosis of GBM patients is abysmal compared to other types [3]. Classical therapy for GBM includes surgical resection, radiotherapy and chemotherapy. However, total surgical resection of GBM is very difficult, thus resulting in a very high chance of recurrence, which directly leads to poor prognosis and seriously impairs patients’ quality of life [42, 43]. Despite new remedies like nanotechnology, stem cell modification and immunotherapy in past decades, the prognoses are still poor [44]. However, studies related to the GBM oncogene are gradually unraveling the pathogenesis of GBM and giving gene therapy a more and more crucial role in the comprehensive GBM treatment [45].

The carcinogenic effects of circRNA have been extensively studied in recent years. A growing number of investigations have identified that circRNAs play vital roles in various cancer occurrences, progression and chemotherapy resistance [46, 47]. Although circRNAs do not directly perform transcription, it is widely confirmed that circRNAs can constitute ceRNAs for exerting corresponding biological effects through miRNA sponging [48]. MiRNAs and circRNAs were critical regulators of mediating tumor growth and oncological behaviors such as invasion, apoptosis, proliferation, and cell metabolism [49]. For example, circPVT1 can promote tumor growth by constituting a sponge of miR-125 in gastric cancer and multiple myeloma [50]. Overexpression of circACP6 can cause the initiation of lung adenocarcinoma via raising the expression of carcinogen cyclin D1 as a result of sponging miR-134 [51]. Therefore, exploring the glioma-related circRNAs, which hold the key to the progression of GBM, is beneficial in guiding the GBM treatment. Our study is the first research that verifies circPTPRF/miR-1208/YY1 axis participates in glioma progression.

We conducted GEO analysis and identified the extremely novel potential oncogene related to GBM progression, circPTPRF. An article has already shown that PTPRF contributes to the development of glioma [52], while a systematic study of how circPTPRF affects GBM progression is still lacking. Hence, there is an urgent need to recognize the biological function and potential mechanisms of circPTPRF in gliomas. Through clinical data analysis and pathological examination of clinical samples, we found that circPTPRF was expressed at higher levels in GBM than in low-grade gliomas and normal tissues. Its expression level was positively correlated with WHO grade and negatively correlated with the survival time of patients.

Then, we further investigated the mechanism related to circPTPRF after proving the situation of cell lines was satisfactory. MTS assays, Edu assays, Transwell assays and NFAs were employed to detect how circPTPRF affected the GBM progressive phenotypes after circPTPRF knockdown and overexpression, respectively. The outcomes demonstrate that the viability, proliferation, invasion capacity, and neurosphere formation capacity of GBM cells were significantly up-regulated when circPTPRF was highly expressed compared with the control group.

After CSCD and circInteractome dataset screening, we found that circPTPRF, as a member of circRNAs, is likely to sponge miR-1208 to promote the development of GBM, and the following LRAs and anti-Ago2 RIP identified this binding between circPTPRF and miR-1208. In previous academic studies, miR-1208 played an oncogenic role in various tumor diseases [24]. Our study found that the expression of circPTPRF was shown to be lower when miR-1208 was at higher expression levels, and the rescue experiments demonstrated that circPTPRF promotes the phenotype of GBM cell lines via the miR-1208-mediated ceRNA mechanism.

Moreover, we found 7 potential targeted genes of miR-1208 according to 4 datasets to further explore the downstream mechanism after illustrating miR-1208 sponging with circPTPRF. We used qPCR to select YY1 as the best candidate gene. LRAs and WB showed that miR-1208 could bind to the 3’UTR of YY1 and negatively regulate YY1 expression. In our subsequent experiments, the expression levels of circPTPRF were shown to have a significant positive correlation with YY1 expression levels. Corresponding rescue experiments also demonstrated that the treatment of YY1 knockdown could reverse the original tumor-promoting effect produced by circPTPRF overexpression. These results indicated that circPTPRF exerts its tumor-promoting effect through the circPTPRF/miR-1208/YY1 axis.

Finally, in the vivo experiment, circPTPRF overexpression significantly increased the tumor size and shortened mice survival time. Immunohistochemistry revealed that the staining intensity of YY1 and ki-67 exceeded that of the control group. circPTPRF knockdown reduced the tumor size and prolonged the survival time of mice, and the staining intensity of YY1 and ki-67 decreased. The data above verified that circPTPRF also had a tumor-promoting effect in xenograft experiments.

This study warrants further exploration, especially the possible reason for circPTPRF dysregulation in GBM. One of the most important reasons for the abnormal expression of circRNA in cancers is the effect of RNA binding proteins (RBPs). RBPs constitute a group of proteins broadly involved in gene transcription and translation. More and more studies showed that RBPs play a vital role in forming and maintaining circRNAs in various cancer [53]. For example, RNA-binding motif protein 20 (RBM20) was related to the structure of a series of circRNA and contributed to forming the class of RBM20-dependent circRNA [54]. Therefore, studying the RBPs targeted to circPTPRF may help further understand the mechanism of circPTPRF in GBM and exert more valuable guidance for clinical treatment.

## Conclusion

Our investigation proved circPTPRF is highly expressed in GBM and is closely related to poor patient prognoses. It promotes GBM malignant biological behaviors by upregulating the expression level of YY1 through the miR-1208 related ceRNA mechanism. Subsequent studies and clinical transformation of circPTPRF are expected to be beneficial in uncovering the pathological mechanisms of GBM and developing clinical treatment options.

## Electronic supplementary material

Below is the link to the electronic supplementary material.

**Supplementary Table S1.** Relationship of circPTPRF expression to clinical features of glioma patients.

**Supplementary Table S2.** Clinical information of the primary glioma stem-like cells.

**Supplementary Figure 1.** The validation of the patient-derived GSCs from fresh clinical GBM specimens. a: Immunofluorescence staining of the stem cell markers CD133+ and Nestin+ in the isolated neurospheres. b: The multi-lineage differentiation capacity and differentiated markers GFAP and ?IIItubulin were shown in the isolated neurospheres. c: The expression of circPTPRF in six GSCs and normal human astrocyte cells (NHA) was detected by qPCR. All data are shown as the mean ? SD (three independent experiments). *P < 0.05; **P < 0.01; ***P  < 0.001.

**Supplementary Figure 2.** CircPTPRF overexpression reversed proliferation and invasion suppression of GBM in vitro caused by circPTPRF knockdown. a, b: MTS assays showed the cell viability of circPTPRF knockdown LN229 and GSC17 after circPTPRF overexpression. c, d: CircPTPRF overexpression can greatly reverse the proliferative capacity of circPTPRF knockdown LN229 and GSC17 measured by Edu assays. Scale bar = 50?m. e, f: Representative images of transwell assay to demonstrate invasion capacity can be reversed after circPTPRF overexpression in circPTPRF knockdown LN229 and GSC17. Scale bar = 50?m. All data are shown as the mean ? SD (three independent experiments). *P < 0.05; **P < 0.01; ***P < 0.001.

**Supplementary Figure 3.** The YY2 expression after miR-1208 mimic or inhibitor treatment in GSCs.a, b: qPCR assays were performed to detect the expression of YY2 in GSC15 after miR-1208 mimic or in GSC17 after miR-1208 inhibitor treatment. c, d: Western blotting showed the expression of YY2 in GSC15 after miR-1208 mimic or in GSC17 after miR-1208 inhibitor treatment. All data are shown as the mean ? SD (three independent experiments). *P < 0.05; **P < 0.01; ***P < 0.001.

## Data Availability

The analyzed data sets generated during the present study are available from the corresponding author on reasonable request.

## List of abbreviations

NcRNAs: non-coding RNAs
ceRNAs: competing endogenous RNAs
miRNAs: microRNAs
GBM: Glioblastoma multiforme
NPCs: neural progenitor cells
GSCs: Glioma stem cells
MREs: microRNA response elements
GEO: Gene Expression Omnibus
H&E: hematoxylin and eosin
IHC: Immunohistochemistry
qRT-PCR/qPCR: Real-Time Quantitative Reverse Transcription PCR
RISC: RNA-induced silencing complex
