# Supplementary material for: Circular RNA circPTPRF promotes the progression of GBM via sponging miR-1208 to up-regulate YY1
Source: Cancer Cell Int. 2022 Nov 17;22:359. doi: 10.1186/s12935-022-02753-1 (PMC9673286; doi:10.1186/s12935-022-02753-1)
Supplement: Supplementary file 1 — Supplementary Table S1. Relationship of circPTPRF expression to clinical features of glioma patients. [file 12935_2022_2753_MOESM1_ESM.docx]

**Table S1.** Relationship of circPTPRF expression to clinical features of glioma patients.

| **Clinical features** | | **Samples**  **(*n* = 55)** | **circPTPRF expression** | | ***P* value** |
| --- | --- | --- | --- | --- | --- |
|  |  |  | **Low (*n* = 25)** | **High (*n* = 30)** |  |
| Sex | Male | 29 | 13 | 16 | 0.921 |
|  | Female | 26 | 12 | 14 |  |
| Age | ≤ 50 | 28 | 11 | 17 | 0.349 |
|  | > 50 | 27 | 14 | 13 |  |
| WHO  grade | II | 15 | 12 | 3 | 0.002 |
|  | III | 20 | 9 | 11 |  |
|  | IV | 20 | 4 | 16 |  |

CircPTPRF expression was detected by qPCR. High expression was defined as higher expressed than the median one while low expression was defined as lower expressed than the median one.
