# Supplementary material for: Circular RNA circPTPRF promotes the progression of GBM via sponging miR-1208 to up-regulate YY1
Source: Cancer Cell Int. 2022 Nov 17;22:359. doi: 10.1186/s12935-022-02753-1 (PMC9673286; doi:10.1186/s12935-022-02753-1)
Supplement: Supplementary file 2 — Supplementary Table S2. Clinical information of the primary glioma stem-like cells. [file 12935_2022_2753_MOESM2_ESM.docx]

Supplementary Table 2. Clinical information of the primary glioma stem-like cells

|  | GSC07 | GSC09 | GSC13 | GSC15 | GSC17 | GSC19 |
| --- | --- | --- | --- | --- | --- | --- |
| Gender | Male | Male | Female | Female | Female | Male |
| Age | 42 years old | 77 years old | 50 years old | 46 years old | 51 years old | 39 years old |
| Location | Left insula | Right parietal lobe | Left parietal lobe | Left parietal lobe | Right occipital lobe | Rght insula |
| WHO grade | IV | IV | IV | IV | IV | IV |
| Ki-67 | 50% (+) | 60% (+) | 40% (+) | 40% (+) | 70% (+) | 60% (+) |
